# Laparoscopic Splenectomy for Sclerosing Angiomatoid Nodular Transformation of the Spleen after a Whipple Procedure: A Case Report

**DOI:** 10.70352/scrj.cr.26-0138

**Published:** 2026-06-25

**Authors:** Kazuki Kato, Hironori Hayashi, Takahiro Yoshimura, Kaichiro Kato, Hiroki Kitabayashi, Masato Hayashi, Daisuke Fujimori, Koichiro Sawada, Masanori Kotake, Kaeko Oyama, Takuo Hara, Shintaro Yagi

**Affiliations:** 1 Department of Surgery, Kouseiren Takaoka Hospital, Takaoka, Toyama, Japan; 2 Department of Hepato-biliary-pancreatic and Transplant Surgery, Kanazawa University Hospital, Kanazawa, Ishikawa, Japan

**Keywords:** laparoscopic splenectomy, pancreatoduodenectomy, sclerosing angiomatoid nodular transformation, Whipple procedure

## Abstract

**INTRODUCTION:**

Sclerosing angiomatoid nodular transformation (SANT) of the spleen is a rare benign vascular lesion. Because histopathological confirmation is required for a definitive diagnosis, splenectomy is typically necessary. We report a case of laparoscopic splenectomy (LS) for splenic SANT in a patient with a history of Whipple procedure for pancreatic serous cystadenoma.

**CASE PRESENTATION:**

A woman in her 40s presented with a splenic mass 6 months after undergoing a Whipple procedure for a serous cystadenoma of the pancreatic head. Contrast-enhanced CT (CE-CT) revealed a 10-mm low-density, space-occupying lesion in the spleen. Twelve months after the Whipple procedure, follow-up CE-CT demonstrated interval growth of the lesion to 14 mm. Fluorodeoxyglucose PET (FDG-PET) showed increased FDG uptake within the mass. LS was performed for complete resection and definitive diagnosis. Histopathological examination confirmed SANT. The postoperative course was uneventful, and there was no evidence of recurrence 12 months postoperatively.

**CONCLUSIONS:**

We report a rare case of splenic SANT detected after a Whipple procedure and successfully treated with LS to exclude malignancy. Further studies are warranted to establish reliable noninvasive diagnostic approaches for SANT.

## Abbreviations

CA19-9
: carbohydrate antigen 19-9
CEA
: carcinoembryonic antigen
CE-CT
: contrast-enhanced CT
EBV
: Epstein–Barr virus
FDG
: fluorodeoxyglucose
FDG-PET
: fluorodeoxyglucose PET
ICG
: indocyanine green
IgG4
: immunoglobulin G4
IPMN
: intraductal papillary mucinous neoplasm
IPT
: inflammatory pseudotumor
LGEA
: left gastroepiploic artery
LS
: laparoscopic splenectomy
OPSI
: overwhelming post-splenectomy infection
SANT
: sclerosing angiomatoid nodular transformation
SCA
: serous cyst adenoma
SOL
: space-occupying lesion
SSPPD
: subtotal stomach-preserving pancreaticoduodenectomy

## INTRODUCTION

SANT of the spleen is a rare benign vascular lesion characterized as a specific, tumor-forming, non-neoplastic process, also referred to as multinodular hemangioma.^1)^ Most patients with SANT are asymptomatic, and the condition is often diagnosed incidentally. Because histopathological confirmation is required for a definitive diagnosis, splenectomy is typically necessary. With recent advances in surgical techniques, LS has become the preferred approach.^2)^ However, a history of upper abdominal surgery, such as a Whipple procedure, is considered a major limiting factor for LS due to postoperative adhesions. We report a case of successful LS for splenic SANT in a patient with a prior Whipple procedure performed for pancreatic serous cystadenoma.

## CASE PRESENTATION

**Fig. 1:**
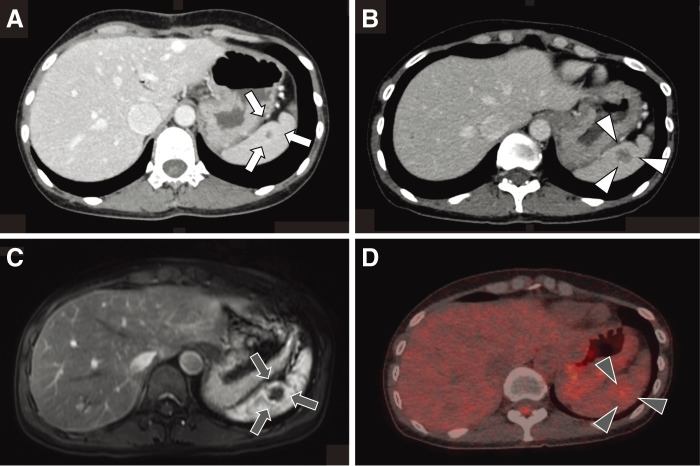
Imaging findings of a progressively enlarging splenic mass following a Whipple procedure. CE-CT performed 6 months after the Whipple procedure revealed a low-density splenic SOL measuring approximately 10 mm in diameter (white arrows) (**A**). At 12 months postoperatively, follow-up CE-CT showed that the lesion had increased in size to 14 mm (white arrowheads) (**B**). CE-MRI demonstrated a ring-enhancing lesion measuring 18 mm (gray arrows) (**C**). FDG-PET revealed FDG uptake within the mass, with a maximum standardized uptake value of 4.24 (gray arrowheads) (**D**). CE-CT, contrast-enhanced CT; FDG, fluorodeoxyglucose; SOL, space-occupying lesion

A woman in her 40s was referred to our department at her first visit for evaluation of a cystic tumor in the pancreatic head that was suggestive of an IPMN, with high-risk stigmata on imaging studies. She underwent SSPPD. Imaging prior to surgery did not reveal any lesions in the spleen. Intraoperative findings also failed to detect any neoplasm. No anti-adhesion agents were administered during the Whipple surgery. Postoperative pathological examination revealed a serous cystadenoma. The patient’s postoperative course was uneventful, and she underwent regular follow-up, including imaging studies. Six months post-surgery, CE-CT identified a low-density SOL in the spleen measuring 10 mm in diameter (**Fig. 1A**). After being informed of these findings, the patient opted for continued surveillance. At 12 months postoperatively, follow-up CE-CT demonstrated enlargement of the splenic SOL to 14 mm, appearing as a low-density mass (**Fig. 1B**). CE-MRI showed a ring-enhancing splenic SOL measuring 18 mm; however, the lesion was not clearly visualized on either T1- or T2-weighted images (**Fig. 1C**). FDG-PET demonstrated FDG uptake within the mass, with a maximum standardized uptake value of 4.24 (**Fig. 1D**). Laboratory findings, including tumor markers such as CEA and CA19-9, were within normal limits. Differential diagnoses included SANT, IPT, lymphoma, and other splenic tumors with malignant potential. Because malignancy could not be excluded due to FDG accumulation, LS was planned for the complete resection of splenic SOL after obtaining informed consent. An ICG fluorescence imaging system was prepared intraoperatively to assess remnant stomach blood flow. In the event of inadequate perfusion after splenectomy, total gastrectomy with Roux-en-Y reconstruction was planned as a contingency.

**Fig. 2:**
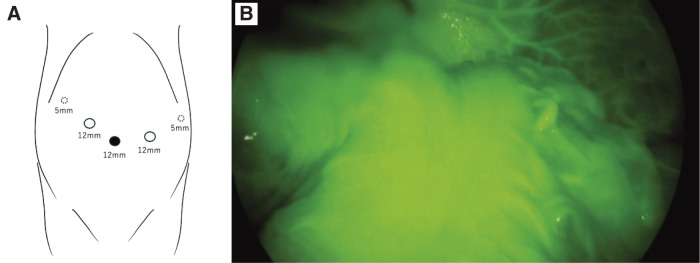
Surgical findings of LS. A 12-mm port was placed in the umbilical region as a camera port, and two 12-mm ports and two 5-mm ports were placed in the upper abdomen (**A**). After splenectomy, the ICG fluorescence method was used to confirm that the blood flow in the residual stomach was maintained (**B**). ICG, indocyanine green; LS, laparoscopic splenectomy

The operation was performed in the supine position using 5 ports (**Fig. 2A**). Intraoperatively, no significant adhesions were observed in the left upper abdominal cavity. After mobilization of the spleen from surrounding structures, the splenic vessels were carefully divided at the splenic hilum distal to the branching of the LGEA. Following splenectomy, adequate arterial pulsation of the LGEA was confirmed, and the ICG fluorescence method was also used to confirm good blood flow preservation in the residual stomach (**Fig. 2B**). Operation time was 161 min, and intraoperative blood loss was minimal.

**Fig. 3:**
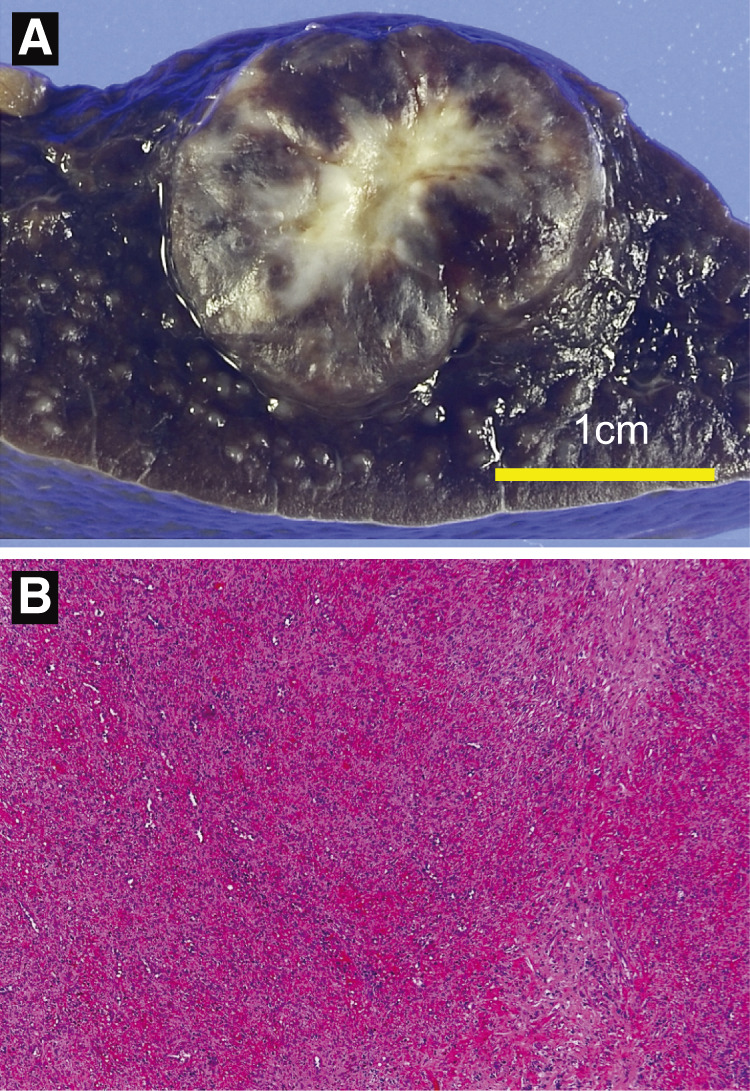
Macroscopic and histological findings of the lesion. The spleen contained a firm, dark reddish nodular mass measuring 15 × 15 mm without a capsule (**A**). Microscopically, the mass was composed of multiple vascular structures separated by fibrous connective tissue (**B**).

**Fig. 4:**
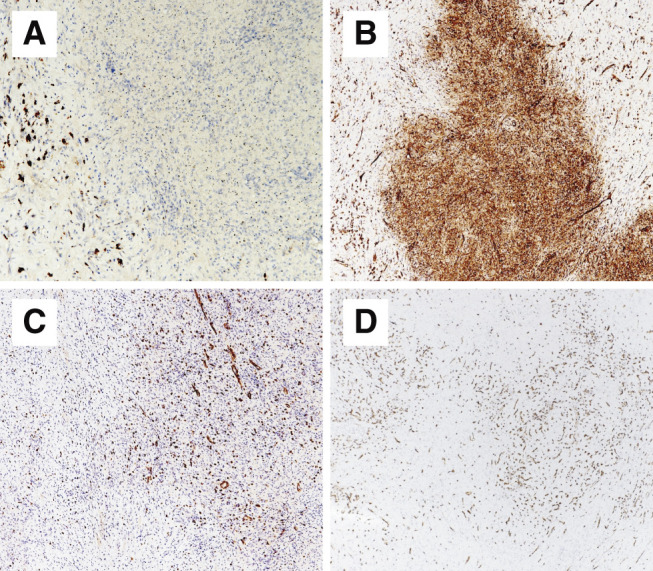
Immunohistochemical staining of the lesion. The tumor cells were positive for IgG4 (**A**), CD31 (**B**), and CD8 (**C**), and negative for CD34 (**D**). IgG4, immunoglobulin G4

Histopathological examination confirmed the diagnosis of splenic SANT. Macroscopically, the spleen contained a firm, dark-red nodular mass measuring 15 × 15 mm. On sectioning, the lesion appeared as a firm, dark-reddish nodule without a capsule (**Fig. 3A**). Microscopically, the mass consisted of multiple vascular nodules separated by fibrous connective tissue (**Fig. 3B**). Immunohistochemical staining demonstrated positivity for IgG4, CD31, and CD8, and negativity for CD34 (**Fig. 4A**–**4D**), consistent with SANT. The patient’s postoperative course was uneventful, with no upper gastrointestinal symptoms. She was discharged on POD 8 without complications, and no evidence of recurrence was observed at 12 months of follow-up.

## DISCUSSION

The diagnosis of splenic tumors is challenging because the spleen has traditionally been regarded as a neglected organ in routine clinical practice, and primary splenic diseases are relatively rare.^3)^

SANT is a benign hemangioma of the spleen first described by Martel et al. in 2004. Its etiology is unclear. SANT is typically identified in middle-aged adults, with a slight female predominance.^1,4)^ Most patients are asymptomatic, and lesions are often detected incidentally during imaging studies or unrelated medical procedures. When symptoms are present, abdominal pain or discomfort is the most common presentation. Aziret et al. reviewed 230 reports from 2004 to 2020 and reported comorbidities including malignant tumors located in tissues other than the spleen (colorectal and gastric cancers) and benign diseases of the gallbladder.^5)^ SANT often presents with a low density of SOL in the spleen, and CE-CT may show a “spoke wheel pattern” in which radioisotope labeling reveals vascular hyperplasia and fibrosis extending from the tumor center to its margins. MRI images are also typically T2-weighted, with low-intensity and delayed central staining.^6)^ Pathologically, a SANT tumor presents as a circular nodule composed of white fibrous tissue. Tissue histology presents a consistent picture with multiple hemangioblastoma nodules in all cases. No recurrence has been reported in patients who have undergone splenectomy.^1)^

The differential diagnosis of splenic SOL is broad and includes many benign and malignant conditions. Cystic lesions, such as congenital true cysts and pseudocysts, are generally easy to diagnose using conventional modalities. In contrast, solid splenic lesions are more difficult to characterize accurately based on imaging alone.

The increasing use of surveillance imaging has led to a growing number of incidentally detected splenic lesions, particularly during postoperative follow-up of patients with prior malignancies or routine health examinations. While SANT normally presents as a circular mass with clear margins, 10% of cases have displayed multiple lesions.^7)^ In this setting, newly identified splenic lesions must be differentiated from metastatic disease. Because SANT can increase in size, it is frequently misdiagnosed by imaging studies as metastasis of a previous malignancy and consequently resected. Previous reports have described suspected primary malignancies, including colon cancer, rectal cancer, and uterine clear cell carcinoma.^8–10)^ As in this case, no reports related to SCA have been found. Despite advances in diagnostic imaging techniques, preoperative diagnosis of SANT remains difficult.

Imamura et al. reviewed the imaging characteristics of SANT, including FDG uptake, and suggested that PET-CT may aid in accurate diagnosis.^6)^ They reported that SANT typically demonstrates low and heterogeneous FDG uptake; however, these findings do not reliably distinguish SANT from malignant splenic tumors. In the present case, PET-CT was performed for further evaluation, but malignancy could not be completely excluded preoperatively. Nevertheless, definitive diagnosis generally requires histopathological examination, especially in patients with a history of neoplastic disease.^11)^

Pathologically, SANT is characterized by angiomatoid nodules composed of spindle cells, inflammatory infiltrates, and proliferating endothelial cells.^12)^ Three distinct immunophenotypic patterns have been described.^13)^ The first, cord capillary–like type, consists of well-formed cord capillaries arranged in lobular patterns and expresses CD34 and CD31 but not CD8. The second type resembles splenic sinusoids and expresses CD31 and CD8, but not CD34. The third type consists of small veins arranged in a complex mesh-like pattern and is positive for only CD8. In the present case, histological findings were consistent with the second type.

SANT has also been reported to be associated with IgG4-related sclerosing disease.^14,15)^ In the present case, immunohistochemical analysis demonstrated positive IgG4 staining, supporting a possible association between SANT and IgG4-related pathology. Kuo et al. reported that the pathological findings of SANT showed an increased ratio of IgG4-positive cells to IgG-positive cells.^14)^ IgG4-related sclerosing disease is a systemic fibroinflammatory condition characterized by dense infiltration of IgG4-positive plasma cells and T-lymphocytes, affecting multiple organs,^16)^ including the pancreas, bile ducts, gallbladder, salivary glands, retroperitoneum, kidneys, lungs, and prostate. However, Nagai et al. reported no other IgG4-related diseases in patients with SANT. In contrast, it has been reported that the average age of patients with SANT is 48.4 years, and that of patients with other IgG4-related diseases is 68.2 years.^15)^ In this patient, serum IgG4 levels were not measured, and there was no history of pancreatitis or cholangitis. However, SANT may be associated with IgG4-related diseases. We believe that SANT cases, such as the present case, should be followed up with attention to IgG4-related diseases after resection. While some reports suggest a link between IgG4-related diseases and EBV infection, to date, no clear causal relationship has been proven.^17)^ EBV infection was not found in this case. Therefore, the clinical relationship between SANT and IgG4-related sclerosing disease remains unclear. However, in cases with imaging findings suggestive of SANT, confirmation of IgG4 and EBV infection may aid diagnosis.

When performing a splenectomy after a Whipple procedure, careful surgical planning is essential to preserve adequate blood flow to the remnant stomach. After the Whipple procedure, gastric arterial supply primarily depends on the left gastric artery and the splenic artery. During splenectomy, the short gastric and left gastroepiploic arteries are often divided during splenic arterial dissection, potentially compromising gastric perfusion. However, residual gastric arterial blood supply is generally preserved, as reported in patients undergoing distal pancreatectomy after distal gastrectomy.^18)^ In the present case, both arteries were preserved through meticulous dissection at the splenic hilum. In addition, intraoperative assessment of gastric perfusion using ICG fluorescence imaging was prepared as a safety measure in the event that splenic artery dissection became necessary. If inadequate perfusion of the remnant stomach had been detected, a completion gastrectomy would have been considered. No reports to date have found an association between gastrointestinal surgery or highly invasive procedures such as Whipple surgery and SANT development.

Splenectomy is generally required for the diagnosis and treatment of splenic SOLs. However, this procedure is sometimes associated with severe complications, including OPSI.^19)^ Jin et al. reported that partial splenectomy is an effective therapeutic approach for SANT.^20)^ Partial splenectomy may also reduce the risk of complications related to asplenia. Percutaneous biopsy has also been reported as a potential method for obtaining tissue samples for the diagnosis of SANT.^8)^ Preoperative splenic biopsy is considered safe, with a low risk of complications such as bleeding; however, there remains a risk of tumor dissemination in the cases of malignant splenic disease.^21)^

Currently, no reliable imaging modality, including FDG-PET, can definitively diagnose SANT. Therefore, surgical resection remains necessary for both diagnosis and treatment.

## CONCLUSIONS

Here, we present a rare case of splenic SANT occurring after a Whipple procedure, which was treated with LS to exclude malignancy. Further studies are warranted to establish reliable noninvasive diagnostic approaches for SANT.

## DECLARATIONS

### Availability of data and materials

All data generated or analyzed during this study are included in the published article.
